# Suicide in a rural area of coastal Kenya

**DOI:** 10.1186/s12888-018-1855-z

**Published:** 2018-08-29

**Authors:** Mary A. Bitta, Ioannis Bakolis, Symon M. Kariuki, Gideon Nyutu, George Mochama, Graham Thornicroft, Charles R. J. C. Newton

**Affiliations:** 1 KEMRI-Wellcome Trust Research Programme, Centre for Geographic Medicine Research Coast, P O Box 230, Kilifi, (80108) Kenya; 2 Health Service and Population Research Department and Institute of Psychiatry, Psychology and Neuroscience, King’s College London, London, UK; 3 Department of Psychiatry, University of Oxford, Oxford, UK

**Keywords:** Suicide, Kenya, Incidence, Risk factors, Verbal autopsy, Demographic surveillance system

## Abstract

**Background:**

Suicide accounts for approximately 1.4% of deaths globally and is the 15th leading cause of death overall. There are no reliable data on the epidemiology of completed suicide in rural areas of many developing countries, yet suicide is an indicator of the sustainable development goals on health.

**Methods:**

Using data collected between 2008 and 2016 from the Kilifi Health and Demographic Surveillance System in rural Kenya, we retrospectively determined the incidence rate and risk factors for completed suicide.

**Results:**

During the period, 104 people died by suicide, contributing to 0.78% (95% CI = 0.74–1.10) of all deaths. The mean annual incidence rate of suicide was 4.61 (95% CI = 3.80–5.58) per 100,000 person years of observation (pyo). The annual incidence rate for men was higher than that of women (IRR = 3.05, 95% CI = 1.98–4.70, *p* < 0.001) and it increased with age (IRR = 2.73, 95% CI = 2.30–3.24, *p* < 0.001). People aged > 64 years had the highest mean incidence rate of 18.58 (95% CI = 11.99–28.80) per 100,000 pyo. Completed suicide was associated with age, being male, and living in a house whose wall is made of scrap material, which is a proxy marker of extreme poverty in this region (OR = 5.5, 95% CI = 4.0–7.0, *p* = 0.02). Most cases (76%) completed suicide by hanging themselves. Spatial heterogeneity of rates of suicides was observed across the enumeration zones of the KHDSS.

**Conclusions:**

Suicide is common in this area, but the incidence of completed suicide in rural Kenya may be an underestimate of the true burden. Like in other studies, suicide was associated with older age, being male and poverty, but other medical and neuropsychiatric risk factors should be investigated in future studies.

## Background

Across the world, one person dies by suicide every 40 s [1]. Suicide accounts for approximately 1.4% of deaths globally and is the 15th leading cause of death overall, and the second leading cause among 15–29 year olds. Studies have shown a link between suicide and mental and physical health, which may have motivated the inclusion of suicide as an indicator for Sustainable Development Goals (SDG) (target 3.4) whose aim is to reduce premature mortality from non-communicable diseases. According to the World Health Organization’s (WHO) 2014 World Report on Suicide, approximately 75% of suicides occur in low and middle-income countries. Unfortunately, the number of studies on the epidemiology of suicide in low and middle income countries is inversely proportional to the burden of mortality from suicide in these regions [2] while sociodemographic determinants of completed suicide have been studied mainly in high income countries [3–5].

The WHO has called upon its member states to create a national strategy on suicide prevention, supported by data which are culturally sensitive [1]. There have been efforts towards this imperative by low and middle income countries such as Kenya which have initiated campaigns to decriminalize suicide with the aim of encouraging suicide attempters to seek help [6]. However, such efforts may result in underreporting of suicide cases in Kenya in fear of prosecution. In 2014, Kenya’s mortality rate due to suicide ranked 29th worldwide with an estimate of 6.5 per 100,000 deaths [7]. However, the reliability of this estimate is unknown because data on deaths by suicide are not systematically collected in Kenya. Additionally, suicide related acts are illegal in Kenya according to penal code 226 of section 36 of the Kenyan constitution hence many deaths may go unreported.

Anthropological studies on the socio-cultural beliefs about suicide in Kenya have been conducted in some ethnic communities such as the Nandi sub-tribe of the Kalenjin tribe [8] and the findings suggest that there are many differences in the beliefs about suicide even among people who share cultural beliefs. Additionally, studies on prevalence and risk factors for suicidal ideation and suicidal gestures whose aim is not death (parasuicide) have been undertaken [9], but so far, there are no reliable studies from Kenya on rates of completed suicide. Kenya is yet to establish an active suicide surveillance system and therefore its estimates of completed suicides may not be reliable. This lack of data impedes planning of suicide prevention strategies.

Risk factors for suicide are numerous and complex [10, 11] and they may vary by community and settings. Identifying modifiable risk factors may be useful in providing knowledge that may inform suicide prevention strategies to target those in most need of care. Suicide may be preventable because known risk factors such as mental illness, if managed appropriately, may lower the risk of suicide, which is feasible because available evidence suggests that people with suicidal thoughts often give warning signs before attempting suicide [1, 12, 13].

We aimed to provide the much-needed reliable estimates of suicide to inform planning of suicide prevention efforts in Kenya. The overall objective of the study was to estimate the incidence rates due to suicide and associations with known socio-demographic risk factors within the Kilifi Health and Demographic Surveillance System (KHDSS) in Kilifi, Kenya.

## Methods

### Study population

**Fig. 1:**
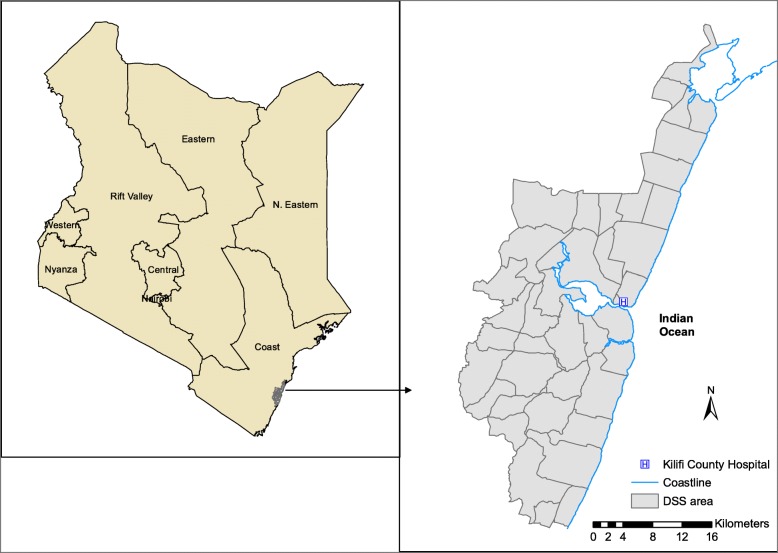
A map of the Kilifi Health and Demographic Surveillance system in the context of Kenya

The study was a cohort analysis of demographic and clinical surveillance data accumulated between the years 2008–2016. The KHDSS is nested in Kilifi County, a rural coastal area along the Indian Ocean in Kenya (Fig. 1). It was established in the year 2000 and has approximately 280,000 residents who form approximately 23% of the entire population in Kilifi County. It contains records of pregnancies, births, migration events, hospital admission events and deaths and is maintained by 4-monthly household visits. Although the entire population participates in routine vital statistics data on births, deaths and migration patterns, only relative or kins of those who died are asked to participate in the verbal autopsy. From 2008, WHO’s Verbal Autopsy Questionnaire has been used to collect data on mortality events in the KHDSS [14]. An extensive description of the KHDSS including the methods of data collection, ascertainment and quality control is provided elsewhere [15].

### Procedures

The main outcome of this study was the mean annual incidence rate of death by suicide. The secondary outcomes were the sociodemographic inequalities of completed suicide. All people living within the KHDSS who died by intentional self-harm were documented to determine the annual incidence rate of suicide,. The socio-demographic inequalities of completed suicide were determined by comparing sociodemographic factors between cases of suicide and participants who died of any other causes. The sociodemographic factors included sex, age, level of education and proxies of extreme poverty such as roofing and wall materials. The cause of death was determined using data from the WHO’s verbal autopsy questionnaire [16], which classifies diseases according to the International Classification of Diseases and Related Health Problems, Tenth Revision (ICD-10). There are 3 versions of the questionnaire depending on the age of the deceased person i.e. under 4 weeks, between 4 weeks to 14 years and above 14 years. The respondents were close family members or relatives of the deceased person. To determine the method of completing suicide, responses to open ended questions in the verbal autopsy questionnaire were examined. All deaths for which verbal autopsy questionnaires were incomplete or missing were regarded as non-suicide deaths.

### Statistical methods

We calculated incidence rate using the mid-year population estimates of person years of observation (pyo) as the denominator and provided the 95% CIs based on a Poisson distribution. To determine the associations of completed suicide with socio-demographic we modelled suicide vs versus non-suicide deaths whereby suicide was coded as “1” and non-suicide deaths as “0” and carried out univariable and multivariable analysis using logistic regression models to obtain odds ratios and their corresponding 95% confidence intervals. Variables with a significance level of *p* < 0.1 in the univariable model were included in the multivariable model, taking care of multicollinearity. Distribution of discreet variables were compared using Pearson’s Chi-squared test or the Fisher’s exact test (in cells with infrequent observations). Strengthening the Reporting of Observational studies in Epidemiology (STROBE) guidelines were used to report the findings of this study [17]. To assess for spatial autocorrelation between the cases of suicide, we used adjacency as a criterion for nearness and calculated the Moran’s I statistic then used robust standard errors to correct for any autocorrelation.

## Results

### Sociodemographic characteristics and morbidity data of people who died by suicide

Between 2008 and 2016, 13,316 people within the KHDSS died, of whom 104 were suicide cases, contributing to 0.78% (95% CI = 0.74–1.10) of all deaths in the KHDSS during this period. Verbal autopsy reports were completed for 11,451(86%) deaths. The median age in years of the suicide cases was 45.5 (IQR = 28.9–60.5) while that of those who died of other causes was 42.0 (IQR = 4.92–67.12). Majority of those who died by suicide were male (*n* = 76, 73.1%). Data on level of education was only collected on a single survey in 2011 and majority (*n* = 96, 92.3%) of the cases did not participate in this survey. Of those who participated, most had never attended school (*n* = 5, 62.5%). Among those who died, no case of suicide was ever admitted to hospital from 2008 to their time of death, whereas 11,184 (84.7%) people who had died of other causes had been admitted to hospital at least once. However, data from the verbal autopsy questionnaire about other medical conditions was available for 96 (90.7%) cases and indicated that high blood pressure occurred in 6 (6.3%), diabetes (*n* = 4, 4.2%), asthma (*n* = 2, 2.1%), epilepsy (*n* = 0), malnutrition (n = 0), cancer (n = 4, 4.2%), tuberculosis (n = 4, 4.2%), HIV/AIDS (*n* = 6, 6.3%) and any other medically diagnosed illness (mental illness, *n* = 1,1.0%). There was no co-occurrence of more than one illness in any case. When asked to describe the illness that led to the deceased person’s death (suicide cases), 20 (18.9%) respondents reported symptoms similar to those observed in psychotic disorders such as hallucinations, delusions and loss of orientation to time and place prior to death. Alcohol abuse was reported in 2 (1.9%) cases and stress related to family issues such as conflicts in marriage and family disputes was reported in 10 (9.4%) cases. The distribution of other sociodemographic factors is shown in Table 1.

**Table 1:** Socio-demographic characteristics and associated risk factors of people who died in the KHDSS between the years 2008–2016

	Completed suicide cases *n* = 104	Non-suicide deaths *n* = 13,212	OR univariable analysis (95% CI)	*p* value	OR multivariable analysis (95% CI)	*p* value
Sex, male	76 (73.08)	6846 (51.91)	3.05 (1.98–4.71)	< 0.001	3.67 (2.35–5.75)	< 0.001
Median age in years (IQR)	45.45 (28.90–60.49)	41.95 (4.92–67.12).	1.04 (1.04–1.06)	< 0.001	1.05 (1.04–1.06)	< 0.001
Roof type						
Metal	25 (24.04)	3632 (27.54)	REF			
Concrete	0 (0)	11 (0.08)	–	–	–	–
Tiled	2 (1.92)	106 (0.80)	2.28 (0.54–9.61)	0.263	2.41 (0.56–10.27)	0.235
Makuti	70 (67.31)	8509 (64.53)	1.68 (1.07–2.66)	0.026	1.55 (0.84–2.86)	0.165
Other^a^	3 (2.88)	280 (2.12)	2.67 (0.81–8.87)	0.107	1.42 (0.37–5.41)	0.609
No response^b^	4 (3.85)	649 (4.92)	–	–	–	–
Wall						
Brick	23 (22.12)	2966 (22.49)	REF			
Mud	70 (67.31)	9252 (70.16)	1.36 (0.85–2.18)	0.200	1.03 (0.54–1.99)	0.920
Other^a^	7 (6.73)	317 (2.40)	5.31 (2.27–12.35)	0.000	3.17 (1.17–8.61)	0.023
No response^b^	4 (3.85)	652 (4.94)	–	–	–	–

^a^refers to roofing/wall materials such as scrap plastic/ metal or polythene sheets

^b^the informant did not provide the information or the data was not collected

*IQR* interquartile range

### Incidence rate of suicide

**Fig. 2:**
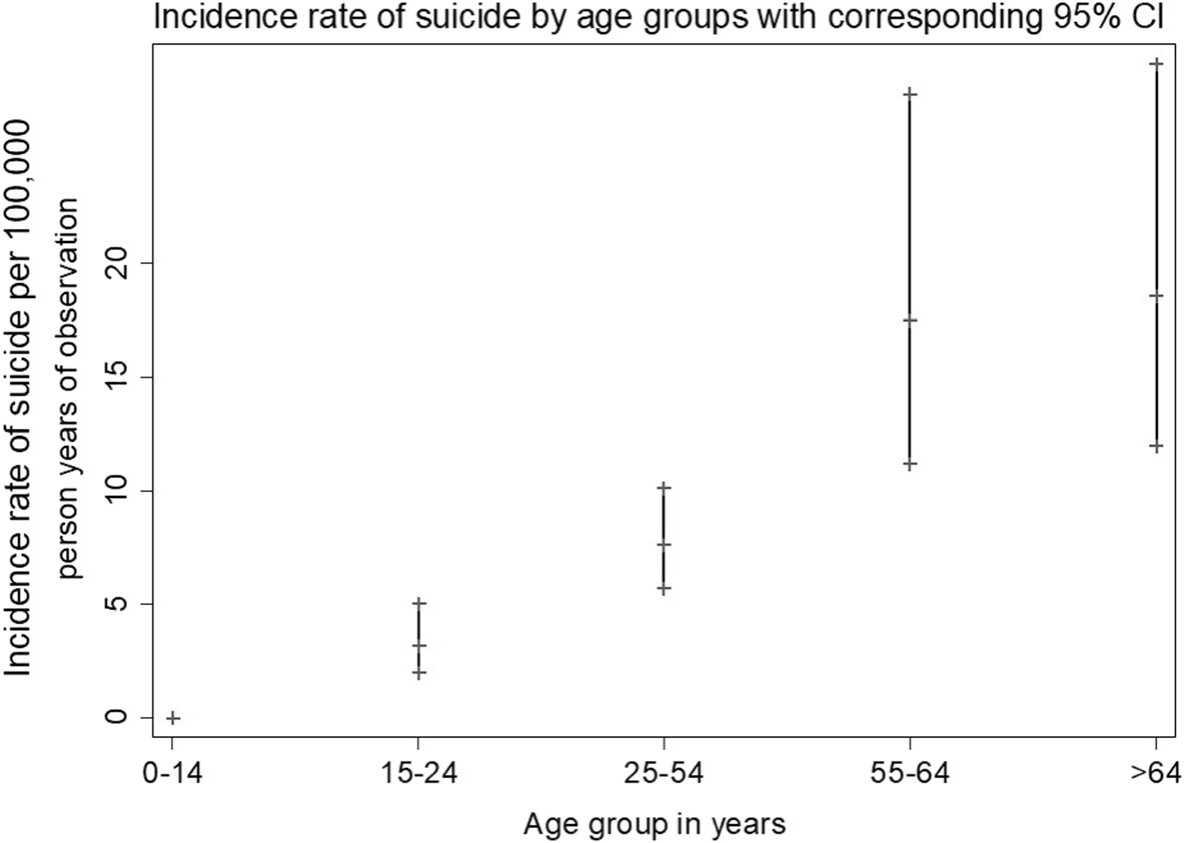
A scatter plot showing the incidence rate of suicide by age groups with the corresponding 95% confidence intervals

The crude mean annual incidence rate of suicide was 4.61 (95% CI = 3.80–5.58) per 100, 000 pyo. Men had a mean crude incidence rate of 7.14 (95% CI = 5.71–8.95) per 100,000, while that of women was 2.35 (95% CI = 1.62–3.40) per 100,000 pyo. The incidence rate for men was significantly higher than that of women (IRR = 3.05, 95% CI = 1.98–4.70, *p* < 0.001). The incidence rate of suicide significantly increased with age (IRR = 2.73, 95% CI = 2.30–3.24, *p* < 0.001) and when separated by age group, the highest incidence rate per 100,000 pyo was observed among those older than 64 years (18.58, 95% CI = 11.99–28.80) as shown in Fig. 2.

**Fig. 3:**
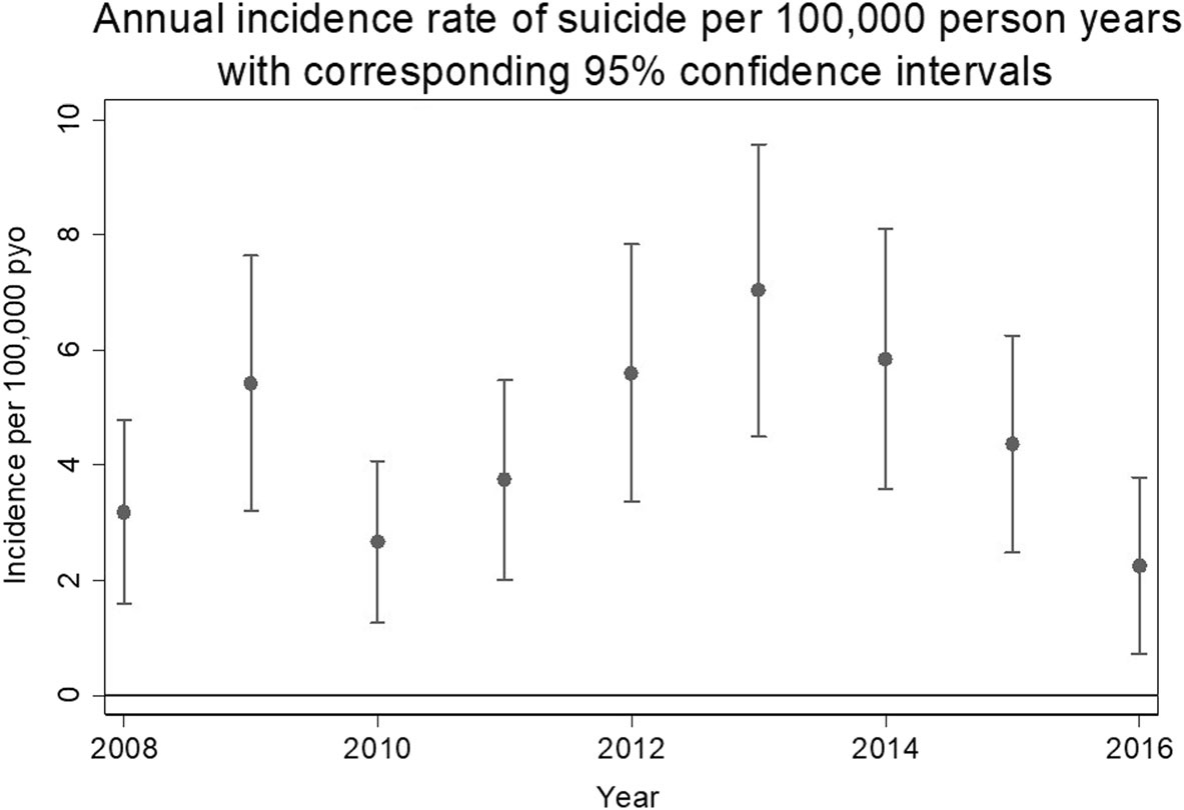
A scatter plot showing annual incidence rate of suicide with the corresponding 95% confidence intervals

The highest incidence rate was observed in the year 2013 (IR = 7.03, 95% CI = 4.49–11.02) and there was no trend in the incidence rates by year (IRR 1.03, 95% CI = 0.95–1.12, *p* = 0.440). Figure 3 shows the annual incidence rate of suicide. Data for the year 2016 were only available up to the month of June and should therefore be interpreted cautiously. There was no spatial autocorrelation between the cases of suicide in the KHDSS (Z = 0.921, *p* = 0.357).

**Fig. 4:**
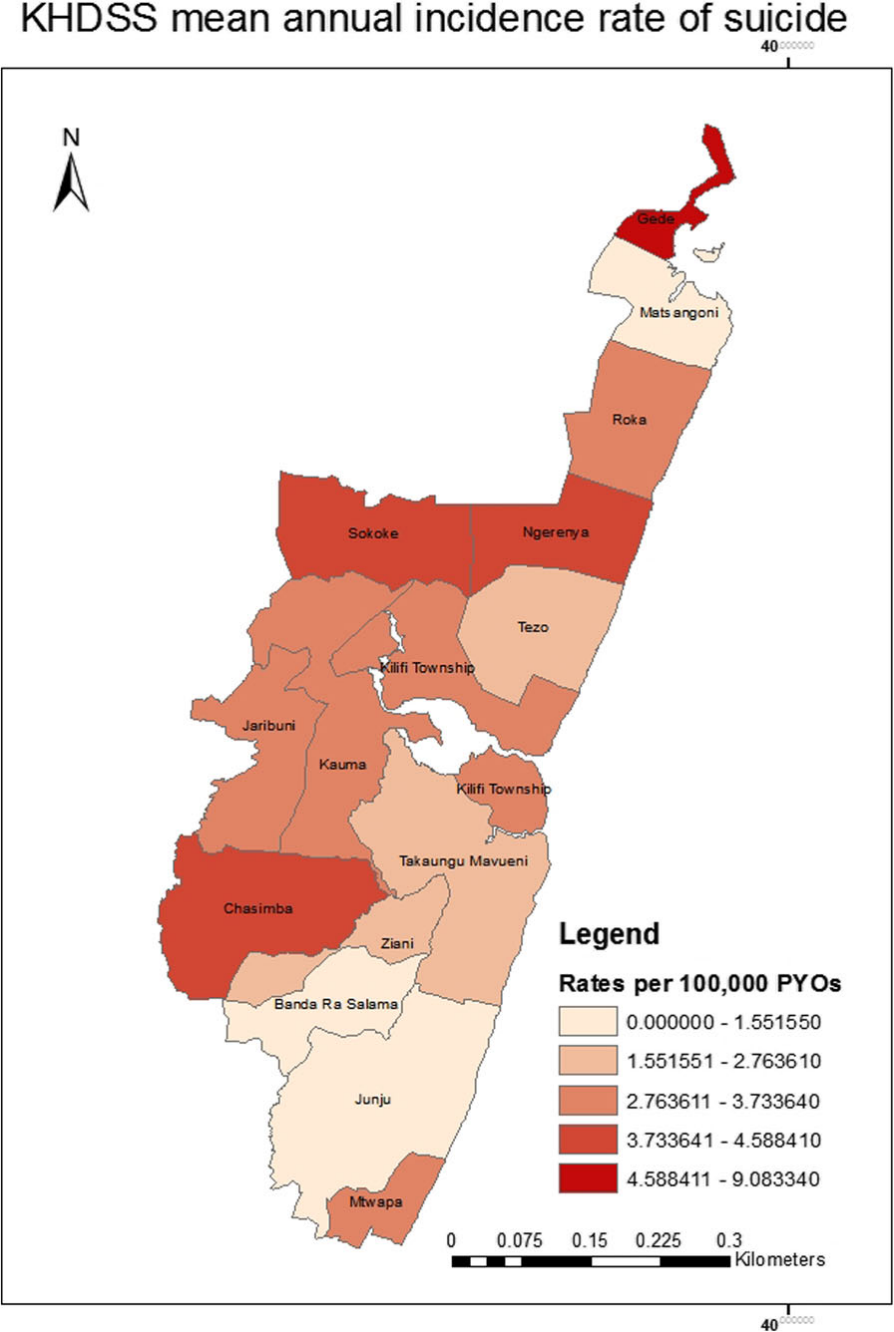
A map showing the mean crude annual incidence rate of suicide by location in the KHDSS

When separated by location, Gede location had the highest crude incidence rate of 21.27 per 100,000 pyo (95% CI = 3.06–154.17) while Matsangoni had the lowest incidence rate of 1.85 (95% CI = 0.93–3.71) as illustrated in Fig. 4.

### Risk factors for and means of completed suicide

In the univariable analysis the odds of death by suicide increased significantly with age (OR = 1.04 95% CI =1.04–1.06, *p* < 0.001, and was higher for males (OR = 3.05 95% CI =1.98–4.71, *p* = 0.000), individuals who lived in houses whose roofs were made of makuti (OR = 1.64, 95% CI =1.04–2.58, *p* = 0.034) and walls were made of scrap materials (OR = 5.31, 95% CI =2.27–12.35, *p* < 0.001) which are markers of extreme poverty in this area. In the multivariable model age, being male and living in house wall made of scrap material was significantly associated with death by suicide as summarised in Table 1.

Data on the means of suicide were available for 66 (62.3%) cases. Majority of the cases (*n* = 50, 76%) hanged themselves using a rope while other means of suicide included self-poisoning (*n* = 9, 14%), intentional drowning (*n* = 2, 3%), intentional drug overdose (*n* = 1, 2%), self-starvation (*n* = 1, 1%) and self-stabbing (*n* = 1, 1%). There were no coroner autopsy or post-mortem data.

## Discussion

To the best of our knowledge, this is the first study that has used longitudinal data from a demographic surveillance system in a low and middle-income country to estimate the incidence rate and socio-demographic determinants of suicide. The results of this study have shown that the mean annual incidence rate of suicide in the KHDSS is 4.61 per 100,000 pyo, accounting for 0.78% of all deaths in the study area. This estimate is lower than the Kenyan average of 6.5, that of lower and middle-income countries of 10.0 per 100,000 [7] and the global average of 1.4%. These estimates are likely to be a minimum and may be greater for the following reasons. Firstly, there was no verification of the suicide reports e.g. through coroner certificates. Secondly, suicide attempts are illegal in Kenya and this may have hindered objective reporting. Thirdly, cultural issues may have reduced reporting for fear of stigma and there may have been misclassification of deaths by other causes such as accidents.

In this study, men had a higher incidence rate compared to women (7.1*vs* 2.4 per 100,000 pyo). This pattern is consistent with other studies [18, 19] and may be because men use more lethal means [11, 20], which result in higher proportions of fatal attempts or because they are less likely to seek help when feeling suicidal because of cultural issues such as gender roles and the definition of masculinity. This is especially plausible for the KHDSS where men are the heads of most households [15].

Analysis of suicide rates by age indicated that older age was associated with completed suicide and when separated by age group, those who were > 64 years had the highest incidence rates. There was also a trend of increasing risk for suicide rate with increasing age. This study showed that although people aged above 64 years form only 4% of the population in Kilifi [21] 42.5% of the suicide cases were from this age group. This may be because of the natural risk of dying with increased age, or because the older population in this study have more risk factors for suicide such as stressful life events like loss of loved ones, as evidenced by the low life expectancy in this setting [21] chronic physical illnesses and the burden of taking care of disabled or orphaned children [22, 23]. Notably the rates among adolescents and young adults aged under 25 were among the lowest. It is uncertain whether this is an artefact of poor reporting or an actual representation of the situation. Data from different parts of the world show a mixed picture of suicide among different age groups. For instance, in Canada, the rates of suicide peak during midlife and they decline thereafter. In the US, suicide rates are a function of sex, age and race where the risk for white men increases continuously with age while that of their black counterparts shows a pattern similar to that of the KHDSS [24]. The similarity in patterns between the black population in the US and the KHDSS population might suggest a genetic link to suicidal tendencies, backing growing evidence on genetic links of suicidal behaviour [25].

The present study found that the most common method of suicide was hanging oneself on a tree. It is possible that this was the easiest form of suicide to identify and report because cases mostly hanged themselves in shared compounds, and therefore many people witnessed the hanging. Although this study did not establish the cause for the preferences of different methods of suicide, a study on survivors of near fatal suicide attempts found that respondents preferred hanging because of the ease of accessing ligature materials and anchor points and because they perceived suicide by hanging to be less painful for them and less traumatizing for those left behind [26]. This poses a challenge to suicide prevention strategies which mainly focus on restriction of means. A few cases also poisoned themselves but it is possible that these cases were under reported because post-mortems are not routinely performed to aid in detecting specific causes of deaths, for instance those who suffocate themselves with poisonous gases. Future post-mortem studies should be carried out to identify the specific substances used in order to understand the best targets for interventions.

An interesting finding from the present study was the lack of formal data on mental illness, despite 19% of the respondents reporting symptoms that mimicked those of a mental disorder in the cases prior to their deaths. We have documented the lack of mental health services in this area [27] which may explain the lack of data on mental illnesses among the cases, and the fact that none of the cases linked up with the clinical database that mostly comprise non-psychiatric problems. The lack of clinical risk factors data for people with mental disorders is critical in explaining the treatment gap for mental health disorders, or inequity of services for mental health problems compared to other somatic/medical problems. Additionally, people with mental illnesses are excluded from routine enumeration exercises mainly because of stigma or because there are no epidemiological studies of mental illnesses such as psychosis in this community. There is evidence of a strong link between mental illness and suicide [20, 28–30], but this study could not quantify the strength of this association because data on the spectrum of mental illness were lacking.

This study also found significant associations between proxy markers of extreme poverty and completed suicide which is similar to findings from a review on suicide and poverty in low and middle income countries [31]. However, this finding should be interpreted cautiously because data available from this study were not sufficient to create a composite measure of socio-economic status that would have been more meaningful in understanding socio-economic position. This study could not establish whether this is a modifiable risk factor, because it lacked qualitative data on the motivation for suicide among the cases. Lastly, there is a need to explore the regional differences in incidence rates observed within the locations of the KHDSS.

### Limitations of the study

This study highlights limitations of the WHO verbal autopsy questionnaire as a method of collecting morbidity and mortality data, and the KHDSS as a method of monitoring vital health statistics. The verbal autopsy questionnaire lacked questions on mental illness as a chronic illness, which limited the conclusions on the risk factors for suicide in this setting. Incomplete or missing verbal auropsy questionnaires were classified as non-suicide deaths which may underestimate the burden of suicide. A locally developed psychological autopsy tool may be more appropriate for studying suicide especially when measuring frequency of suicide as a comorbidity of other conditions**.** The data relied on a retrospective account of events leading to the death, which may have introduced recall bias. Additionally, data on previous attempts of suicide, which is the strongest predictor of completed suicide, were lacking. The verbal autopsy questionnaire is not administered to all cases of death in this setting mainly because of lack of appropriate respondents. For instance in this study and in a previous study on all causes of death in this setting, the questionnaire was not administered to 14% of all deaths [32]. This may further underestimate the suicide cases.

## Conclusion

Suicide is common in this rural area of Kenya, but the incidence rates of suicide may have underestimated the true burden. This study could not establish whether low socio-economic status is a modifiable risk factor for completed suicide and there were no sufficient data to examine the role of mental illness in the risk of suicide. Early identification and mitigation of known risk factors such as mental illnesses and other clinical risk factors may be a way of preventing suicide later in life in low and middle income countries. Additionally, future analyses on the epidemiology of suicide should include clinical risk factors. There is room for better utilization of the KHDSS as a resource of health monitoring and improvement of the WHO verbal autopsy questionnaire to capture vital data on mental illnesses.

## Abbreviations

HIV/AIDS: Human Immunodeficiency Virus/ Acquired Immune Deficiency Syndrome
ICD-10: International Classification of Diseases and Related Health Problems, Tenth Revision
KHDSS: Kilifi Health and Demographic Surveillance System
SDG: Sustainable Development Goals
STROBE: Strengthening the Reporting of Observational studies in Epidemiology
WHO: World Health Organization
